# Prevalence, risk factors and molecular characterization of Cryptosporidium infection in cattle in Addis Ababa and its environs, Ethiopia

**DOI:** 10.1016/j.vprsr.2018.03.005

**Published:** 2018-08

**Authors:** Anberber Manyazewal, Stomeo Francesca, Mahendra Pal, Mamo Gezahegn, Mulatu Tesfaye, Muthui Lucy, Wegayehu Teklu, Tilahun Getachew

**Affiliations:** a Department of Microbiology, Immunology and Vet. Public Health, College of Veterinary Medicine and Agriculture, Addis Ababa University, P.O. Box 34, Debrezeit, Ethiopia; b The Biosciences Eastern and Central Africa-International Livestock Research Institute (BecA-ILRI) Hub, P.O. Box 30709, Nairobi, Kenya; c Department of Parasitology, National Animal Health Diagnostic and Investigation Centre(NAHDIC), P.O. Box04, Sebeta, Ethiopia; d Collage of Natural Sciences, Arba Minch University, P.O. Box 21, Arba Minch, Ethiopia; e Aklilu Lemma Institute of Pathobiology, Addis Ababa University, P.O. Box 1176, Addis Ababa, Ethiopia

**Keywords:** *Cryptosporidium*, Prevalence, Risk factors, PCR-RFLP, Ethiopia

## Abstract

A cross-sectional study was conducted to determine the prevalence and risk factors of *Cryptosporidium* infection and identify species of the parasite in cattle in central Ethiopia. Faecal samples, collected from 392 dairy cattle managed under intensive and extensive production system, were analyzed by the Modified Ziehl-Neelsen (MZN) microscopy, Nested PCR, PCR-RFLP and sequence analyses of the SSU rRNA gene of *Cryptosporidium*. The overall prevalence, the prevalence in the extensive and intensive farms was 18.6%, 11% and 21%, respectively. The infection was detected in 37.7% of the investigated farms with prevalence range of 7.4% -100%, and all of the six surveyed districts with significant (*P* = 0.000) prevalence difference. Restriction digestion and sequence analysis showed *Cryptosporidium parvum* and *C. andersoni* in 27% and 73% of the infections, respectively, showing an age related distribution pattern, *C. parvum* exclusively occurring in calves <2 months old and *C. andersoni* only in heifers and adult cattle. The infection was significantly associated with management system, farm location, herd size, source of drinking water, weaning age, presence of bedding, pen cleanness and cleanness of hindquarter. In conclusion, *Cryptosporidium* infection due to *C. parvum* and *C. andersoni* was prevalent in cattle in the study area. *Cryptosporidium parvum* has the concern of public health importance, especially to farm workers and people in close contact with cattle. Instigation of imperative control measure is suggested to lessen the risk of human infection and loss of production in dairy farms.

## Highlights

• *Cryptosporidium* infection, due to *C. parvum* and *C. andersoni,* is widespread in cattle in central Ethiopia.
• *C parvum* and *C andersoni* exclusively occurred in calves <2 months and in adult cattle, respectively, showing an age related distribution pattern.
• Risk factors of *Cryptosporidium* were mainly associated with management and hygienic condition of animals.
• existence of the zoonotic *C parvum Spp,*alerts instigation of urgent preventive/control measures.
• Molecular tools are crucial to characterize *Cryptosporidium* species

## 1 Introduction

*Cryptosporidium* infection in livestock may cause important economic impact to farmers due to its high morbidity and sometimes, high mortality rates among farm animals (Casemore et al., 1997). Four species: *C. parvum*, *C. bovis*, *C. andersoni* and *C. ryanae* are commonly found affecting cattle (Feng et al., 2007; Brook et al., 2008). *C. parvum* is generally associated with diarrhoea in susceptible hosts causing illness and even death, particularly in neonatal calves (Plutzer and Karanis, 2009). Infected farm animals, particularly cattle, are considered to be sources of human infection. This concern has put pressure on researchers and farmers to identify and manage the risks associated with spread of the zoonotic infection. The design of strategic plan to control infections in target population depends largely on understanding the factors that lead to the introduction, transmission, and spread of infection in animals (Mohammed et al., 1999). Epidemiologic studies that consider the multi-factorial nature of diseases will provide valuable information to prevent the occurrence and spread of infection in animals and ultimately reduce public health concern (Mohammed et al., 1999).

In Ethiopia, studies on cryptosporidiosis in dairy farms are scarce yet; the few studies conducted to date reported prevalence rates ranging from 7% to 27.8% (Abebe et al., 2008; Adamu*,* 2010; Alemayehu et al., 2013; Wegayehu et al., 2013; Dinka and Berhanu, 2015; Wegayehu et al., 2016), and signified the importance of dairy cattle to human infection. Most of these studies employed conventional microscopy which is unable to characterize species of the parasite. Therefore, the aim of this study was to determine the prevalence and risk factors of the infection and to characterize species of the parasite infecting cattle in Addis Ababa and its surrounding rural districts.

## 2 Materials and methods

### 2.1 Study area

The study was conducted in 69 randomly selected dairy farms and Peasant Associations (PAs) found in six districts (Akaki Kality, Dukem, Sebeta, Barak, Holota and Sululta) in Addis Ababa and Oromia regional states, central Ethiopia.

### 2.2 Sampling

A cross-sectional study design was used to select 392 animals residing in 69 dairy farms and PAs using the stratified random sampling technique. Faecal samples, collected directly from the rectum of animals, were put into ice box and transported to the laboratory where each sample was portioned in to two halves. The first half was examined by the modified Ziehl-Neelsen (MZN) staining technique within two days of collection while the remaining half was preserved in 2.5% potassium dichromate solution and kept at +4 °C.for molecular studies.

### 2.3 Questionnaire and data collection

A pretested questionnaire comprising mainly close ended questions was used to generate information on herd-level hypothesized risk factors for *Cryptosporidium* shedding including management system, farm location, herd size, weaning age, existence of calving pen, method of colostrum feeding, presence of bedding, source of drinking water, disposal of farm waste water, access to water, pen type and presence of other diseases. Farm inspections made on sampling dates was used to collect data requiring personal observation such as sanitation and cleanness of animals.

### 2.4 Laboratory examinations

#### 2.4.1 Direct microscopy of faecal specimen

The Modified Ziehl-Neelsen (MZN) staining technique (Henriksen and Pohlenz, 1981) was used to detect and identify oocysts of *Cryptosporidium* under the 100× magnification of the microscope. A sample was considered positive if an oocyst of correct morphology, optical properties, internal structure, size and shape (4–6 μm, refract pink, spherical round to oval with a residuum and sporozoites) was detected (Fayer, 1997).

#### 2.4.2 Isolation of genomic DNA

Genomic DNA was extracted from 56 oocyst positive to 144 randomly selected oocyst negative faecal specimens. Prior to DNA extraction, 200 mg (100–200 μl) of each sample was washed three times in distilled water and centrifuged to clear out the preservative solution. DNA was extracted using the QIAamp DNA Stool Mini Kit (QIAGEN inc. Valencia, USA) following the manufacturer's suggested procedures and was stored at −20 °C until further analysis.

#### 2.4.3 Nested PCR

A two-step nested PCR protocol was used to amplify a fragment of the SSU rRNA gene of *Cryptosporidium* species oocyst (840 bp) as described previously (Fayer and Xiao, 2008). In the primary PCR, a PCR product of 1325 bp was amplified using the forward and reverse primers SSU-F2 (5′-TTCTAGAGCTAATACATGCG-3′) and SSU-R2 (5′-CCCATTTCCTTCGAAACAGGA-3′), respectively. The primary, 25 μl, PCR mixture consisted of 1× PCR buffer, 6 mM MgCl_2_, 0.2 mM of each of the four deoxyribonucleotide triphosphates (dNTPs), 10 pmol of each primer, 2.5 units of Taq DNA polymerase (Roche Diagnostics, Mannheim, Germany), 5 μl of template DNA and 0.5 μl of non-acetylated bovine serum albumin (BSA; 10 mg/ml) (New England Biolabs, Beverly, MA, USA). The Primary PCR cycling conditions consisted of an initial denaturation at 94 °C for 3 min, followed by 35 cycles (94 °C for 45 s, 55 °C for 45 s and 72 °C for 1 min) with a final extension at 72 °C for 7 min and cooling at 4 °C. In the secondary PCR, a product size of 826 to 864 bp (depending on isolates) was amplified using the forward primer SSU-F3: 5′-GGAAGGGTTGTATTTATTAGATAAAG-3′and the reverse primer SSU-R4: 5′-CTCATAAGGTGCTGAAGGAGTA-3′ (Xiao et al., 1999, Xiao et al., 2001; Fayer and Xiao, 2008). The secondary PCR reaction mixture consisted of 1× PCR buffer, 3 mM MgCl_2_, 0.2 mM of each dNTPs, 10 pmol of each primer, 2.5 units of Taq DNA polymerase and 2 μl of the primary PCR product in a final volume of 25 μl. The cycling conditions for the secondary PCR were the same as the primary PCR. Secondary products of the nested PCR reaction were analyzed by 1.5% agarose gel electrophoresis and visualised on Gel red staining. All PCR reactions were run on a Thermocycler (PCR-Gene Amp PCR System 9700, Applied Biosystems) and DNA concentration was determined spectrophotometrically on a Nanodrop 2000 (Thermo Fisher Scientific inc.USA).

#### 2.4.4 Restriction fragment length polymorphism (RFLP) analysis

The secondary PCR products of the 18S rRNA gene were purified using the QIAquick PCR purification kit (QIAGEN) according to the manufacturer's instruction and were digested using the *SspI* or *MboII* (New England Bio Labs Inc.) restriction enzymes (Xiao et al., 1999, Xiao et al., 2001; Feng et al., 2007). Briefly, 10 μl of the purified secondary PCR product was digested with 5 units of enzyme and 2 μl of the corresponding 10× buffer in a final volume of 20 μl. All restriction digestions were carried out at 37 °C overnight, fractionated on 2% agarose gel and visualised after Gel red staining.

#### 2.4.5 DNA sequencing

The sequencing reaction of purified secondary nested PCR products was performed in both directions using the secondary PCR primers and the Big Dye Terminator V3.1 Cycle sequencing Kit (Applied Biosystems, CA, U.S.A.) on an ABI 3730-48 Capillary Genetic Analyzer (Applied Biosystems Sequencer, Foster City, CA).The obtained sequences were analyzed using the CLC main workbench (CLC version 7.6.4, QIAGEN Aarhus) and compared with the Gene Bank sequences of *Cryptosporidium* using BLAST (Basic Local Alignment Search Tool, NCBI http://www.ncbi.nlm.nih.gov/blast/Blast.cgi) to identify species and determine homology percent. Nucleotide sequence data reported in this paper are available in the GenBank database under the accession numbers KX264360 to KX264365. Sequencing was performed at the Segolip unit, the Biosciences Eastern and Central Africa-International Livestock Research Institute Hub (BeCA-ILRI Hub), Nairobi, Kenya.

### 2.5 Statistical analysis

Data were analyzed by the SPSS statistical software package (SPSS ver.20.0 for Windows, SPSS Inc., Chicago, IL).The GLM Univariate Analysis of Variance and the Chi-Square test were used to evaluate the bivariate association between hypothesized factors and the risk of *Cryptosporidium*. Association of the risk factors with infection rate were further analyzed using the logistic regression analysis and the effect of each factor on the likelihood of *Cryptosporidium* infection was quantified by the odds ratio (OR) which was computed as the exponent of the respective regression coefficient. Confidence level was held at 95% and P < 0.05 was set for significance level.

## 3 Results

### 3.1 Prevalence of *Cryptosporidium*

*Cryptosporidium* was obtained in 73 of the 392 samples examined with an overall prevalence of 18.6%. Twenty six of the sixty nine investigated dairy farms and PAs, and all of the six surveyed districts had shown the infection with significant (P = 0.000) prevalence difference among the districts (Table 1). The prevalence in the intensively managed cattle, 21.4%, was significantly (P = 0.021) higher than the 11.2% prevalence in the extensively managed animals. Dairy farms sited in urban area showed significantly (P = 0.000) higher prevalence than farms located in rural area. Prevalence of the infection was similar across the age groups (P = .749) and between the sexes (P = 0.062) (Table 1). Animals under intensive farms and animals in farms sited in urban area were 2 times (OR = 2.156, 95% CI = 1.11–4.180) and 4.5 times (OR = 4.91, 95% CI = 2.81–8.54) more likely to acquire the infection as compared to animals under extensive management system and animals from farms in rural area, respectively.

**Table 1:** Prevalence of *Cryptosporidium* infection in cattle by Age, sex and management system (June/2014 - Dec /2015) (N = 392).

Variables	Group	No^a^	Prevalence		P value
			Percent	95% CI^b^	
Age group	<2 months	104	17.3	9.8–24.8	0.749
	2–6 months	209	18.2	12.9–23.5	
	>6 months	79	21.5	2.9–30.2	
Sex	Female	253	21.3	16.5–26.1	0.062
	Male	139	13.7	7.2–20.1	
Management system	Extensive	107	11.2	3.8–18.6	0.021
	Intensive	285	21.4	16.9–25.9	
Farm location	Rural	313	12.8	8.6–16.9	0.000
	Urban	79	41.8	33.5–50.0	

Overall		392	18.6	14.75–22.49	

a Number of samples.

b Confidence interval.

### 3.2 Risk factors

Risk factors assessed during this study were categorized into factors related to farm location and management and factors linked to water supply and sanitation. Among the factors related to farm location and management, group penning, medium herd size, absence of calving pen, absence of calf bedding, dam suckling and weaning age ≥6 months showed significant association with increased infection rate (Table 2). Whereas, among the latter category, river/stream water sources, limited access to drinking water, disposal of farm waste water to wells, occurrence of other diseases (Foot and Mouth Disease and Pasteurellosis), unclean pens and unclean tail, hindquarter and flank of animals were significantly associated with increased prevalence of *Cryptosporidium* (Table 3). Animals from farms lacking calving pens and from farms with no practice of calf bedding were 2.5 times (OR = 2.46, 95% CI = 1.08–5.61) and 10.5 times (OR = 10.55, 95% CI = 4.89–22.66) more likely to acquire *Cryptosporidium* as compared to animals from farms with calving pens and farms practicing calf bedding, respectively, (Table 2). Farms holding medium and small herd size were about 7 times (OR = 6.99, 95%CI = 3.32–14.71) and 3 times (OR = 2.93, 95%CI = 1.40–6.13) more likely to catch *Cryptosporidium* compared to farms with larger herd size. Farms where colostrum is hand fed and weaning age is <six months have shown the infection 2 and 3.5 times less likely as compared to farms practicing dam suckling and weaning age ≥6 months, respectively (Table 2).

**Table 2:** Risk factors of *Cryptosporidium* related to general management system in cattle in Addis Ababa and its environs, June 2014–Dec /2015(N = 392).

Risk factors	Label	Prevalence (%)	Adjusted OR		
			OR^a^	95%CI^b^	P value
Management system	Extensive	11.2	2.16	1.1–4.2	0.016
	Intensive	21.4			
Farm location	Rural	12.8	4.89	2.8–8.5	0.000
	Urban	41.8			
Presence of calving pen	Yes	9.6	2.46	1.1–5.6	0.019
	No	20.7			
Method of colostrum feeding	Hand feeding	11.8	2.15	1.2–3.9	0.009
	Suckling	22.3			
Presence of bedding	Yes	4.26	10.55	4.9–22.7	0.000
	No	31.7			
Weaning age	<6 months	17.5	3.45	1.8–6.8	0.001
	≥6 months	42.2			
Herd size	>100	7.4			0.000
	<30	18.9	2.93	1.4–6.1	
	30–100	35.8	6.99	3.3–14.7	

a Odds ratio.

b Confidence interval.

**Table 3:** Risk factors of *Cryptosporidium* related to water and sanitation in cattle in Addis Ababa and its environs, June/ 2014–Dec 2015 (N = 392).

Risk factors	Category	Prevalence (%)	Adjusted OR		
			OR^a^	95%CI^b^	p value
Source of drinking water	Pipe	10.3			0.003
	Well	21.4	2.36	1.2–4.9^b^	
	River/stream	24.8	2.86	1.5–5.5	
Disposal of farm waste water	To a field	14.6	2.79	1.6–4.9	0.000
	To well	32.2			
Access to water	Free access	14.3	2.11	1.1–4.0	0.018
	Limited	26.0			
Presence of other diseases	No	16.4	5.10	2.3–11.6	0.000
	Yes	50.0			
Pen cleanness	Clean	12.4	2.37	1.1–4.9	0.014
	Medium/unclean	25.0			
Cleanliness of hindquarter	Clean	13.1	1.96	1.1–3.4	0.013
	Medium/unclean	22.8			
Pen type	Individual pen	10.4	2.66	1.0–7.0	0.030
	Group pen	23.6			

a Odds ratio.

b Confidence interval.

Neatness of pen and animals' hindquarter had reduced the infection by two folds (OR = 2.0, 95%CI = 1.13–4.93) (Table 3). Farms using wells and river/stream as drinking water sources acquired *Cryptosporidium* 2.4 and 2.9 times, respectively, compared to farms using tap water. Animals having limited-water access acquired *Cryptosporidium* 2 times (OR = 2.11, 95% CI = 1.13–3.96) more likely compared to animals with free-drinking water access. Farms with history of Pasteurellosis and FMD had shown about 5 times (OR = 5.10, 95%, CI = 2.25–11.55) more *Cryptosporidium* as compared to farms without record of these diseases. Decline in *Cryptosporidium* by >2.5 times (OR = 2.79, 95%CI = 1.62–4.91) was seen in farms disposing waste water to the field as compared to farms dumping waste water to nearby wells. Individual sheltering of calves had lessen *Cryptosporidium* infection by about 2.5 times (OR = 2.66, 95%CI = 1.01–7.04) compared to group penning (Table 3).

In contrast to the above findings, the study results showed absence of statistically significant association between *Cryptosporidium* infection and type of barn floor (concrete/soil/stone), method of floor cleaning (dry vs. wet), experience of attendants (≤5 years vs. >5 years), history of diarrhoea, farm age (1–5 years, 6–10 years, 11–30 years), breed (local zebu vs. crossbreed (Holstein Friesian × zebu) and mode of water supply (group vs. Individual).

### 3.3 Molecular results

**Fig. 1:**
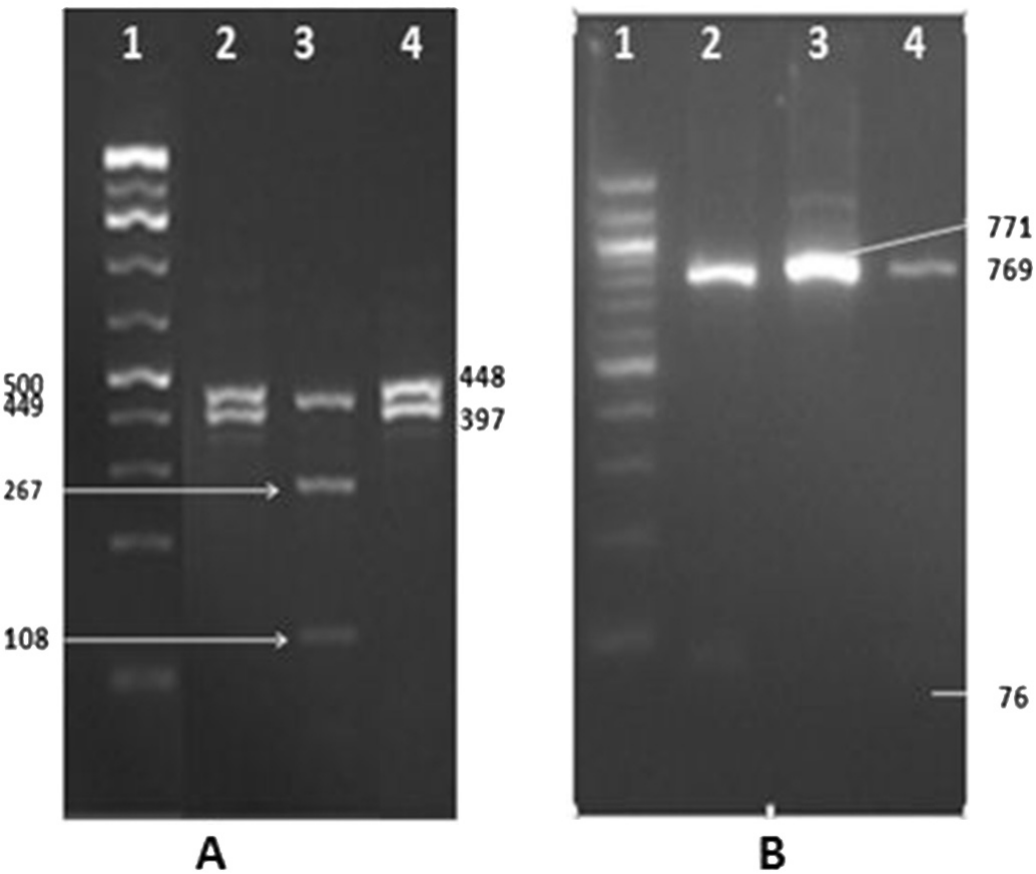
Species identification of *Cryptosporidium* by PCR-RFLP analysis of the 18S rRNA gene. Digestion by *Ssp*I (A) and *Mbo*II (B) restriction enzymes. Lane 1: 100 bp ladder; Lanes 2 & 4: *C. andersoni*; Lane 3: *C. parvum.*

All microscopy positive (56) and 17 of the microscopy negative samples generated the expected PCR product of approximately 830-bp. RFLP restriction of the 73 purified secondary PCR products indicated *C. parvum* in 19 (26.0%) specimens, and *C. andersoni* in 54 (74.0%) specimens (Fig. 1). Electrophoresis of *Ssp*I digested products showed the predicted restriction patterns of *C. parvum* with three visible bands at the level of 449, 267 and 108 bp while *C. andersoni* generated two bands of 448 and 397 bp as described previously (Feng et al., 2007). *Mbo*II digested secondary PCR products showed restriction patterns with two visible bands of 771 and 76 bp for *C. parvum*, and bands of 769 and 76 bp for *C. andersoni* (Fig. 1). *Cryptosporidium parvum* infections were exclusively detected in neonates and <2 month calves, while *C. andersoni* infections were noticed in calves >3 months, heifers and adult cows demonstrating age related distribution of the infection in the study herds. Mixed infections were not detected in this study.

### 3.4 Sequence analysis

Clear sequences of the SSU rRNA gene were obtained from 45 of the 73 isolates. Homology search in the GenBank indicated that 12 of the 45 isolates (26.7%) were *C. parvum* and 33 of the 45 isolates (73.3%) were *C. andersoni*. These results confirmed the identification of *Cryptosporidium* species previously carried out by the RFLP analysis. BLAST (Basic Local Alignment Search Tool) searches of the partial SSU rRNA gene sequences showed 99% similarity to reference sequences in the GenBank for *C. parvum* (KP004206, ×64340, S40330 and ×64341) and 100% similarity to reference sequences in the GenBank for *C. andersoni* (KJ917578, AB777193, AB513856, AB089285, HM002493, FJ608606, KM199850, KF826311). (http://www.ncbi.nlm.nih.gov/blast/).

## 4 Discussion

The overall prevalence of *Cryptosporidium* infection in cattle obtained in this study, 18.6%, was comparable to the prevalence of 17.6% reported in central Ethiopia (Abebe et al., 2008), lower than the prevalence report of 27.8% by Alemayehu et al. (2013) and higher than the 7.8%, 13.6% and 15.8% prevalence reports by Wegayehu et al. (2013), Dinka and Berhanu (2015) and Wegayehu et al. (2016), respectively. Studies conducted in other parts of the world also stated varied prevalence rates: comparable values of 18.8%, and 17% were reported by Budu-Amoako et al. (2012) and Keshavarz et al. (2009), respectively. Higher prevalence rates ranging from 27% to 86.7% had been reported (Santin et al., 2004; Nguyen et al., 2007 and Venu et al., 2012) and lower values of 12% and 11.7% were reported by Hamnes et al. (2006) and Khan et al. (2010), respectively.

The difference in the overall prevalence of *Cryptosporidium* among different studies could be due to variations in ecology, study design, season, management system, age, herd size and laboratory techniques employed. Animals reared under intensive management system were more affected by *Cryptosporidium* (21.4%) than those under the extensive system (11.2%) which could be due differences in breeds of animals as well as confinement, higher stocking rate and crowding in the intensive dairy farms favouring more contamination of barns, high contact of animals and rapid dissemination of oocysts compared to extensive farms. In the semi-intensive or intensive management system of rearing animals are confined to a restricted area, thus continuously contaminated the surroundings (Maikai et al., 2011). This result is in agreement with the findings of Geurden et al. (2006) that reported prevalence of 42.8% for animals reared under intensive system and 6.3% for animals under extensive system. Comparable lower prevalence had been reported in extensive farms compared to intensive farms (Ralston et al., 2003; Santin et al., 2004). Disposal of farm waste water to ground wells was associated with increased risk of *Cryptosporidium* compared to disposal to distant fields. Contamination of farm water or feed store by the nearby waste-water wells might be the reason for the observed difference as this site favours survival of oocysts; conversely, disposal of farm waste-water over wider field area may exposé oocysts to high environmental temperature and desiccation for which Oocysts are susceptible (Li et al., 2010). Reduction in viability and infection pressure has been reported due to dispersal of oocysts over larger surface that exposes them to direct sunlight (Li et al., 2010). Although *Cryptosporidium parvum* is considered a cause of diarrhoea in neonates and many previous studies reported significant association between diarrhoea and shedding (Geurden et al., 2006; Karanis et al., 2010), no association was detected in this study which might be due to differences in pathogenicity of the strains or co-infections by other diarrhoea causing enteropathogens that could mask the effect of *Cryptosporidium*. Similar results showing absence of association between diarrhoea and shedding had been reported (Abebe et al., 2008; Rieux et al., 2013). Animals from farms without provision calf bedding acquired more *Cryptosporidium* compared to animals from farms with provision of calf bedding. Since daily appliance and disposal of calf bedding has major effect in lessening oocyst persistence in pens, this could explain the reduced contamination of farms and lower infection prevalence in animals for which calf bedding is provided. Maddox et al. (2006) stated that addition of clean bedding and its daily disposal had significantly declined the risk of *Cryptosporidium* infection. Other studies also showed that bedding and hygiene related factors had significant effect on the odds of infection (Castro-Hermida et al., 2006; Brook et al., 2008b). Significant association was observed between the infection and group penning as well as weaning age ≥6 months. Both risk factors could lead to sound contact and likely transmission of the infection from young calves to neonates. Calf-to-calf contact is suggested to be the most likely route of transmission, and averting of this tends to decrease the infection (Kvác et al., 2006).

*Cryptosporidium* was significantly associated with absence of calving facilities and practice of dam suckling; higher chance of infection might have resulted due to exposure of neonates to their dams or other group of the herd in farms where calving facilities are absent, or if newborns stayed with their dams in maternity pens in case of farms with calving facilities. Our result is in agreement with findings that reported higher prevalence of the disease in newborns due to their closeness with their dams (Del Coco et al., 2008; Santin et al., 2008; Bjorkman et al., 2015). *Cryptosporidium parvum*-like oocysts were detected at two days of age and *C. parvum* was confirmed by molecular analysis at an age four days indicating transmission of oocysts either from the dam or from contamination of calving pens (Silverlås, 2010). The present study illustrates that infections were significantly higher in farms with previous record of Foot and Mouth Disease or Pasteurellosis compared to farms without these diseases. These diseases are highly infectious and known to cause severe illness with immune suppression effect, thus it is likely to find higher prevalence in such farms since *Cryptosporidium* is an opportunistic parasite severely affecting immunocompromized animals (Fayer and Xiao, 2008).

Increased risk of *Cryptosporidium* was seen in farms using river/stream water sources which could be due to their higher exposure to the environment and contamination by faeces of humans, domestic and wild animals. Reports indicate that river water is heavily contaminated with oocyst of *Cryptosporidium* in proportion to the number of cattle in the adjacent area and livestock waste were more pollutant of river water compared to sewages (Yang et al., 2008). Results of this study showed that animals having unclean hindquarters and/or housed in unclean pens showed higher infection rates than animals with clean hindquarters and/or housed in clean pens. Favourable conditions created by the wet and soiled floors may possibly help oocyst survival and spread of the infection among animals. Our results are in accord with the findings of Abebe et al. (2008) that reported a 5.2 times odds of infection in calves housed in poorly cleaned farms compared to calves in well-cleaned farms. Zhang et al. (2013) and Maddox et al. (2006) highlighted significant association between daily cleaning of pens and reduction in the risk of *Cryptosporidium* infection. Differing from earlier studies that reported higher risk of infection in larger herds (Silverlås, 2010; Inpankaew et al., 2014) our study demonstrated lower infection rate in larger herds. The reason for this finding might be the better sanitary practice noticed in larger farms, mainly managed by professionals, compared to medium and small sized farms run by owners or non professional personnel.

All isolates from calves <2 months genotyped by PCR-RFLP of the *Ssp*I and *Mbo*II restriction enzymes in this study were identified as *C. parvum*. Whereas, isolates from older calves (>2 months), heifers and adults generated restriction pattern typical for *C andersoni*. The restriction enzyme *Mbo*II is indicated for the easy differentiation of the common *Cryptosporidium* spp. in cattle, Its use in conjunction with *Ssp*I RFLP facilitate rapid genotyping of all four common Cryptosporidium spp. in cattle (Feng et al., 2007). *Mbo*II digestion of the secondary SSU rRNA PCR products would generate two bands for *C. parvum* (76 and 771 bp), *Cryptosporidium andersoni* would also produce two bands (76 and 769 bp), but it could be easily differentiated from *C. parvum* by *Ssp*I restriction (Feng et al., 2007).

*Cryptosporidium parvum* and *C. andersoni* identified in the current study showed an age related distribution; *C. parvum* was encountered in calves less than two months and *C. andersoni* in older calves, heifers and adult cattle. This finding is in agreement with earlier reports in the country (Adamu, 2010; Wegayehu et al., 2016), and reports in other countries that indicated four species, *C. parvum, C. andersoni, C. ryanae*, and *C. bovis* commonly affecting cattle (Santin et al., 2008; Keshavarz et al., 2009; Silverlas et al., 2010; Rieux et al., 2013). Comparable age related distribution pattern had also been reported by different researchers (Santin et al., 2004; Feng et al., 2007; Plutzer and Karanis, 2007; Thompson et al., 2007; Keshavarz et al., 2009; Liu et al., 2009). In support of this finding, studies on 12–24 weeks and 2–6 month old calves in Nigeria and Vietnam reported absence of the zoonotic *C. parvum* species and suggested that these age groups of calves were unlikely to contribute to human cryptosporidiosis (Ayinmode et al., 2010; Nguyen et al., 2012). *C. parvum* infection rates of 0.4% and 0.7%, was reported in milking cows and heifers, respectively, and it was suggested that yearling and mature dairy cattle are relatively low risk sources of human infections (Fayer et al., 2006, Fayer et al., 2007).

Majority of the *C. parvum* infections in this study were encountered in neonates below one month of age which is in concord with findings of Castro-Hermida et al. (2002) and Santin et al. (2004) that reported occurrence of most *C. parvum* infections between the first and fourth week of life. In addition, Díaz-Lee et al. (2011) and Del Coco et al. (2008) reported the highest *C. parvum* infection in calves 7–14 days and 8–21 days of age, respectively. Contrary to the present finding Castro-Hermida et al. (2007) reported only *C. parvum* in cows, and Wells et al. (2015) reported that 96% of the infections in adult cattle were due to *C. parvum*. The difference in the occurrence and distribution of major species infecting dairy calves suggest that the transmission of *Cryptosporidium* may be different among different herds of cattle (Ma et al., 2015). All isolates from calves <2 months genotyped by PCR-RFLP of the *Ssp*I and *Mbo*II restriction enzymes in this study were identified as *C. parvum*. Whereas, isolates from older calves (>2 months), heifers and adults generated restriction pattern typical for *C andersoni*. The restriction enzyme *Mbo*II is indicated for the easy differentiation of the common *Cryptosporidium* spp. in cattle. Its use in conjunction with *Ssp*I RFLP facilitate rapid. Our result is in agreement with literature data that indicated *C. parvum* as the most frequently found species in pre-weaned calves (Xiao et al., 2002; Santin et al., 2004; Trotz-Williams et al., 2006; Thompson et al., 2007; Feng et al., 2007; Plutzer and Karanis, 2007).

## 5 Conclusion

*Cryptosporidium* infection of cattle due to *C. parvum* and *C. andersoni* was widespread in the study area with an overall prevalence of 18.6%. The magnitude of infection was higher in animals managed under intensive production system than animals under extensive production system. Risk factors associated with *Cryptosporidium* infection were mainly related to general management, farm sanitation and hygiene of animals due to scarcity of water and contamination water sources. Awareness creation on risk factors, sources of infection and means of transmission is imperative for prevention and control of the disease in cattle and humans. Further molecular epidemiology studies covering various parts of the country are recommended to establish allocation of species and national impact of the disease.

## Ethical clearance

Ethical clearance was obtained from the College of Veterinary medicine and Agriculture, Addis Ababa University. The aim of the study was explained and permissions were obtained from farm owners and employees before collection of samples and data.

## Conflict of interest statement

The authors declare that they have no financial or personal relationship(s) that may have inappropriately influenced them in writing this article.
